# Assessment of Ecological Quality of the Tajan River in Iran Using a Multimetric Macroinvertebrate Index and Species Traits

**DOI:** 10.1007/s00267-015-0489-x

**Published:** 2015-04-11

**Authors:** Jaber Aazami, Abbas Esmaili Sari, Asghar Abdoli, Hormoz Sohrabi, Paul J. Van den Brink

**Affiliations:** Department of Environment, Faculty of Natural Resources, Tarbiat Modares University, Tehran, Iran; Department of Biodiversity and Ecosystem Management, Environmental Research Institute, Shahid Beheshti University, Tehran, Iran; Department of Forestry, Faculty of Natural Resources, Tarbiat Modares University, Tehran, Iran; Department of Aquatic Ecology and Water Quality Management, Wageningen University, Wageningen University and Research Centre, Wageningen, The Netherlands; Alterra, Wageningen University and Research Centre, Wageningen, The Netherlands

**Keywords:** Macroinvertebrate multimetric index, Biological water quality, Macroinvertebrates, Traits, Tajan River, Iran

## Abstract

The objectives of this study were to assess the biological water of the Iranian Tajan River using different metrics, i.e., a Multimetric Macroinvertebrate Index (MMI) and a traits-based method. Twenty-eight physico-chemical parameters, 10 habitat factors, and abundance of macroinvertebrates were obtained for 17 sites. The Shahid-Rajaie dam divides the Tajan River into an up- and downstream part, with different land uses. Eighteen metrics were used to represent four components of ecosystem quality, including tolerance (Hilsenhoff, SIGNAL), diversity (Margalef, Shannon–Wiener, Simpson, and Evenness), abundance (total number of taxa, individuals, Ephemeroptera, Plecoptera, Trichoptera, EPT, and Insects), and composition of assemblages (% Ephemeroptera, % Plecoptera, % Trichoptera, and % EPT Taxa). The integrated MMI was calculated by averaging the obtained scores of all indices. In the next step, we gathered information on 22 biological traits of macroinvertebrates to evaluate whether (group of) traits could be identified that are indicative for specific or general stress. Result showed a decrease in MMI from upstream (very good water quality) to downstream (bad) due to human activities. Industrial activities like pulping and papermaking operations or sand mining in the downstream part had more effects than agriculture and fish ponds in the upstream part. A redundancy analysis biplot showed the variation between the modalities of trait of macroinvertebrates and their correlation with physico-chemical parameters in Tajan River. The findings show that traits can be indicative for different kind of stress but that more effort has to be put in gathering data sets to disentangle the effect of habitat quality, pollution, and the physico-chemical properties of high- versus lowland rivers.

**Electronic supplementary material:**

The online version of this article (doi:10.1007/s00267-015-0489-x) contains supplementary material, which is available to authorized users.

## Introduction

Many countries in Asia are located in a semi-dry area with limited water resources. In recent years, the increase in human populations resulted into negative effects on water quality, therefore, necessitating the formulation of a good management strategy of water resources in this area (Hosseini et al. 2009). For a good assessment of the water quality, it is important to include important aquatic biota such as macroinvertebrates. There are many studies that used macroinvertebrate assemblages for assessing the ecological quality of aquatic ecosystems, since they are affected by the physical, chemical, and biological conditions of the stream (Menetrey et al. 2011). They are good indicators of stream health because they cannot escape pollution, and some are more sensitive to pollution than others and can be used as indicators (Barbour et al. 1999; Van Ael et al. 2015). They may show the cumulative impacts of multiple stressors, like habitat loss, which are not always detected by the traditional water quality assessments using physico-chemical measurements. Macroinvertebrates are a critical part of the stream’s food web and they are relatively easy to sample and identify. We used macroinvertebrates for assessing the ecological water quality and the development of a Multimetric Macroinvertebrate Index (MMI) for the Tajan River, Iran.

We also used a European trait database to evaluate the correlations between the physico-chemical parameters and the trait composition to evaluate whether traits could be identified that are specific to certain stressors or indicative for general stress (Culp et al. 2011; Schuwirth et al. 2015). A trait is defined as a characteristic that reflects a species adaptation to its environment. Traits describe the physical characteristics, ecological niche, and functional role of a species within the ecosystem. Traits-based bioassessment uses traits to explain or predict variation in ecological system condition and is now being introduced into the field of Ecological Risk Assessment (ERA) and bioassessment of ecological quality (biomonitoring) of aquatic ecosystems (Van den Brink et al. 2011a; Menezes et al. 2010). Also an analysis of strengths, weaknesses, opportunities, and threats (SWOT) of using macroinvertebrate traits has been published (Van den Brink et al. 2011b).

Some biotic and physico-chemical indices to classify the water quality in Tajan River have been reported by Aazami et al. (2015). The goals of that study were to determine and classify the water quality based on Iranian Water Quality Index for Surface Water Resource-Conventional Parameters (IRWQIsc), National Sanitation Foundation Water Quality Index (NSFWQI), the ratio of Biological Monitoring Working Party score to Average Score per Taxon (BMWP/ASPT), Multimetric Macroinvertebrates Index Flanders (MMIF), Karr Biotic Index of Fish (KBI), Rapid Bioassessment Protocol of Environmental Protected Agency, USA (RBP EPA), and the evaluation of their performance. Also, it includes a Geographic Information System (GIS) analysis to show and assess the effects of human land uses on the Tajan River (Aazami et al. 2015). The objectives of this study are to develop a biological MMI and use macroinvertebrate traits to assess the ecological water quality which is done for the first time for an Iranian river. The index has recently been successfully used for assessing the ecological water quality of a river basin in Vietnam (Nguyen et al. 2014). We also determine the correlation between physico-chemical parameters and ecological traits to evaluate their diagnostic power and, herewith, providing more information about Tajan River to managers.

## Materials and Methods

### Study Area

**Fig. 1:**
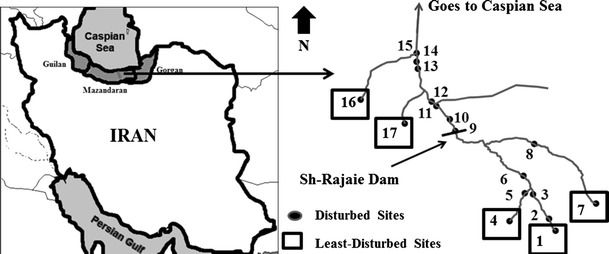
Map of Tajan River and sites in the north of Iran

Data were collected throughout the Trajan basin, which is drained by the Tajan River and located in Mazandaran Province, Iran. This was chosen as a pilot river from the 115 rivers in Northern provinces of Iran (Guilan, Mazandaran, and Gorgan Provinces) because of having a good water flow, discharge regime, catchment area, valuable environmental condition, and different land uses (Fallah and Farajzadeh 2008). The basin area of the river is 140 km long, originates from forested mountains, and continues through the different land uses including agricultural areas of the coastal plain, where rice is extensively cultivated, and finally, it goes to the Caspian Sea, the biggest land-locked aquatic ecosystem in the world (Fig. 1). There are different land uses in the river including agriculture, aquaculture, damming, sand mining, and industrial activities (Namin et al. 2013; Ahmadi-Mamaqani et al. 2011). It is divided into up- and downstream part by an old, large dam (Shahid-Rajaie Dam). For this study, we sampled macroinvertebrates and measured physico-chemical and habitat parameters at 17 sites in September 2013, of which 8 sites are located upstream and 9 sites downstream. Also, five sites were selected as least-disturbed sites (LDS) where there was no or slight pollution expected compared to disturbed sites (DS). Site selection was based on land use, accessibility, and anthropogenic activities.

### Physico-chemical and Habitat Parameters

In this study, 28 physico-chemical and habitat parameters were measured at each site. Width, length, and depth were measured in situ by handheld meters, dissolved oxygen (mg/L), pH, water and air temperature (°C), conductivity (µS/cm), turbidity (NTU), and nutrients (NO3-N, NO2-N, NH4-N, and PO4-P, mg/L) were measured in situ using portable multi-parameter water analyzer and UV–Vis Spectrophotometry 8000 that was provided by Tarbiat Modares University, Iran. Biochemical oxygen demand (BOD mg/L) and total suspended solids (TSS, mg/L) were determined according to protocols set by APHA (Eaton and Franson 2005), with three replicate measurements in the laboratory. Habitat assessment was performed using 10 factors assessed by four experts, and the RBP_EPA_ methodology was used for the river habitat assessment by visual observations at each site (Barbour et al. 1999). The range of each habitat parameters was from 0 (very perturbed) to 20 (pristine).

### Macroinvertebrate Sampling

The benthic macroinvertebrate fauna was sampled using a standard surber sampler (30 × 30 cm and 250-μm mesh) as explained by Tomanova et al. (2008). For each site, three replicates were collected and all the three replicates were composited as one sample. Benthic macroinvertebrates were preserved in 4 % formaldehyde solution before being sorted, identified, and counted to family level in laboratory using available identification keys (Needham and Needham 1962; Fernández and Domínguez 2001).

### Multimetric Macroinvertebrate Index

Eighteen metrics were used to represent four components of ecosystem quality, including tolerance, diversity, abundance, and composition of macroinvertebrate assemblages (Table 1) (Nguyen et al. 2014). We used the Family Biotic Index (FBI) that is described in Hilsenhof (1988) and the Stream Invertebrate Grade Number Average Level (SIGNAL) as described by Chessman (2003) as tolerance metrics. We also included the Margalef diversity index which is calculated from the total number of species present and the total number of individuals. The Shannon–Wiener, Simpson, and Evenness indices were calculated by a free ecological software program: Past, V3.04 (Myers et al. 2000). Finally, the selected metrics were normalized to standardize the variance following the approach by Hering et al. (2006):

$$ {\text{Value}} = \left( {{\text{metric}}\;{\text{result}} - {\text{lower}}\;{\text{anchor}}} \right)/\left( {{\text{upper}}\;{\text{anchor}} - {\text{lower}}\;{\text{anchor}}} \right). $$

**Table 1:** Candidate metrics for the development of Macroinvertebrate Multimetric Index plus their values for the LDS (least-disturbed sites), DS (disturbed sites), and All (all sites) together with the significance of their difference indicated (significance indicated with an asterisk)

Metric category	Abbreviation	Mean			Mann–Whitney *U* test (*P* value)
		LDS	DS	All	
Tolerance					
Hilsenhoff	HBI	3.78	2.44	3.11	0.12
SIGNAL	SIG	4.61	2.65	3.63	0.01*
Diversity					
Margalef	MAR	4.98	4.20	4.59	0.28
Shannon–Wiener	SHA	2.98	2.81	2.90	0.31
Simpson	SIM	0.92	0.89	0.91	0.04*
Evenness	EVE	0.62	0.60	0.61	0.04*
Abundance					
Total number of taxa	TNT	30.20	27.12	28.66	0.00*
Total number of individual	TNI	651.40	346.16	498.78	0.01*
Number of Ephemeroptera	NoE	282.60	95.08	188.84	0.27
Number of Plecoptera	NoP	32.40	43.58	37.99	0.19
Number of Trichoptera	NoT	61.21	42.75	51.98	0.04*
Number of EPT	NEPT	376.20	191.41	283.81	0.03*
Number of Insects	NoI	651.43	364.16	507.80	0.05*
Composition					
% Ephemeroptera		42.47	24.33	33.40	0.01*
% Plecoptera		12.75	4.95	8.85	0.01*
% Trichoptera		12.87	9.19	11.03	0.10
% EPT Taxa		76.42	52.15	64.29	0.09

Lower and upper anchors were the lowest and highest obtained scores per metrics in the monitoring campaign. To develop the final index, the integrated MMI was calculated as the average of the obtained scores, i.e., the sum of all scores ranging from 0 to 1 divided by the number of individual metrics. This results in a final index ranging from 0 to 1 which can be interpreted as an overall ecological quality index. The final MMI was split into five subclasses of equal range with values close to 1 (class 1) indicating the best quality scores as opposed to values near 0 (class 5) (Nguyen et al. 2014).

### Biological Traits

We gathered information on 22 biological traits of macroinvertebrates based on an European trait database provided by Bonada et al. (2011). Each of the modality of traits had subgroups (Table 2); for example, for maximal potential size, there were 7 subgroups including maximal potential size: (1) ≤0.25 cm, (2) >0.25–0.5 cm, (3) >0.5–1 cm, (4) >1–2 cm, (5) >2–4 cm, (6) >4–8 cm, and (7) >8 cm. Trait scores were computed for each family that was sampled by averaging the trait scores of genera present in the database and belonging to the same family.

**Table 2:** Macroinvertebrate trait modalities as extracted from the trait database for the taxa present in the presented data set

Traits name	Abbreviation	Number of modalities	Modalities
Maximal potential size	MaxPotsi	7	≤0.25, 0.25–0.5, 0.5–1, 1–2, 2–4, 4–8, >8 cm
Life-cycle duration	LifCycDu	2	≤1, >1 year
Potential number of cycles per year	NumCycl	3	<1, 1, >1
Aquatic stages	AquSta	4	Egg, larva, nymph, adult
Dispersal	Disp	4	Aquatic passive, aquatic active, aerial passive, aerial active
Resistance forms egg	ResForEg	5	Eggs (statoblasts), cocoons, housings against desiccation, diapause or dormancy, none
Respiration	Resp	5	Tegument, gill, plastron, spiracle, hydrostatic vesicle
Locomotion and substrate relation	LocAndSu	8	Flier, surface swimmer, full water swimmer, crawler, burrower, interstitial, temporarily attached, permanently attached
Food	Food	9	Microorganisms, detritus (<1 mm), dead plant (≥1 mm), living microphytes, living macrophytes, dead animal (≥1 mm), living microinvertebrates, living macroinvertebrates, vertebrates
Substrate (preferendum)	Sub	9	Flags/boulders/cobbles/pebbles, gravel, sand, silt, macrophytes, microphytes, twigs/roots, organic detritus/Litter, mud
Current velocity (preferendum)	CurVel	4	Null, slow (<25 cm/s), medium (25–50 cm/s), fast (>50 cm/s)
Trophic status (preferendum)	TroSta	3	Oligotrophic, mesotrophic, eutrophic
Salinity (preferendum)	Salin	2	Freshwater, brackish water
Temperature (preferendum)	Temp	3	Cold (<15 °C), warm (>15 °C), eurythermic
Saprobity	Sapro	5	Xenosaprobic, oligosaprobic, b-mesosaprobic, a-mesosaprobic, polysaprobic
pH (preferendum)	pH	6	≤4, >4–4.5, >4.5–5, >5–5.5, >5.5–6, >6

### Statistical Analysis

The ShapiroWilk test incorporated in SPSS 19 (licensed by Tarbiat Modares University, Iran) was used to check for normality of the data distribution, and the result was that the data were not normally distributed. Therefore, the non-parametric Mann–Whitney *U* test was used to assess the significance of the differences of the values of the indices between DS and LDS. The Spearman correlation was used to assess the significance of the correlations between abiotic parameters and the biotic indices.

The multivariate technique redundancy analysis (RDA) was used to assess the correlations between traits and abiotic parameters. The taxon-by-sample and taxon-by-trait matrices were combined into a trait-by-sample matrix. To limit the number of explanatory variables in the analysis, only abiotic parameters explaining a significant part (Monte Carlo permutations under the RDA option; *P* ≤ 0.05) of the variation in trait composition between the sites were included in the final RDA analysis performed with the Canoco version 5 program, licensed by Wageningen University, The Netherlands (Ter Braak and Šmilauer 2012).

## Results and Discussion

### Physico-chemical Variables

**Fig. 2:**
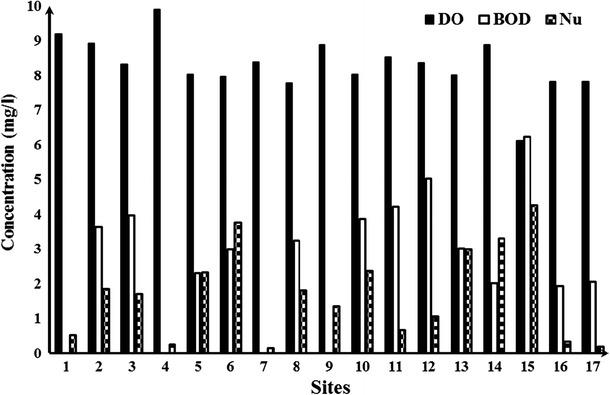
Changes of DO, BOD, and nutrients in sites of Tajan River (for the exact placement of the sites is referred to Fig. 1)

Of all physico-chemical parameters, turbidity and nitrite showed the highest exceedance of the threshold values set by ISIRI, followed by phosphate and ammonium. There was a pronounced difference in water quality between the DS and LDS (Table 3). DO and pH decreased, while turbidity, BOD, nutrients, and TSS increased from up- to downstream (Fig. 2). So all physico-chemical parameters indicated an increase in eutrophication and stress from suspended solids from up- to downstream.

**Table 3:** Summary statistics of abiotic parameters for both sites (least-disturbed and disturbed) in Tajan River, Iran

Parameters	Abbreviation	Mean		ISIRI	
		LDS	DS	*A*	*B*
Dissolved oxygen (mg/L)	DO	8.86	7.52	≥6.00	≥4.00
Hydrogen ion concentration	pH	8.12	7.75	7	6.50–8.50
Turbidity (NTU)	Tur	1.50	73.5	≤1.00	≤5.00
Nitrate (mg/L)	NO_3_	0.25	1.66	3	10
Nitrite (mg/L)	NO_2_	0.51	1.07	0.01	0.04
Ammonium (mg/L)	NH_4_	0.06	0.54	0.5	2
Phosphate (mg/L)	PO_4_	0.11	0.45	–	–
Nutrient (NO_3_ + NO_2_ + NH_4_ + PO_4_, mg/L)	Nu	0.33	2.45	3	10
Biochemical Oxygen Demand (mg/L)	BOD	1.02	8.11	4	15
Total suspended solids (mg/L)	TSS	39	759	1000	1500

ISIRI is the maximum acceptable level based on Institute of Standards and Industrial Research of Iran (*A* for drinking water purposes,* B* for irrigation and transportation or other activities that does not require a high-quality standard)

### Multimetric Macroinvertebrate Index

Based on the MMI, a clear deterioration of the water quality was observed after site 11 from upstream to downstream, with side branches of the downstream stretch having a better quality than the main river (Tables 1, 4). The difference between LSD and SD was expressed significantly by the SIGNAL tolerance index, the Simpson and Evenness diversity indices, the total number of taxa and individuals, and the number of individuals belonging to Trichoptera, EPT taxa, and insects. Also the % of individuals belonging to Plecoptera and Trichoptera differed significantly between the LSD and SD. The fourteen physico-chemical parameters had a correlation with at least one biotic metric as assessed by the spearman correlation analysis. Depth was negatively correlated with 4 of the indices, while turbidity, DO, and ammonium were positively correlated with 3, 4, and 5 indices, respectively. The strongest correlation was between ammonium and the number of Plecoptera (*r* = 0.76) and Shannon–Wiener (*r* = 0.69; Table 5).

**Table 4:** Classes of ecological quality along the Tajan River basin based on the MMI

Up/down	Site	Distance	Category	Ecological quality		
				MMI value	Water class	Interpretation
Upstream						
	1	0	LDS	0.89	Class 1	Very good
	2	1.65	DS	0.59	Class 3	Moderate
	3	3.08	DS	0.63	Class 2	Good
	4	0	LDS	0.81	Class 1	Very good
	5	3.89	DS	0.69	Class 2	Good
	6	2.71	DS	0.57	Class 3	Moderate
	7	0	LDS	0.91	Class 1	Very good
	8	9.78	DS	0.77	Class 2	Good
Downstream						
	9	12.89	DS	0.81	Class 1	Very good
	10	1.29	DS	0.69	Class 2	Good
	11	2.89	DS	0.55	Class 3	Moderate
	12	0.71	DS	0.38	Class 4	Poor
	13	3.54	DS	0.32	Class 4	Poor
	14	0.61	DS	0.16	Class 5	Bad
	15	1.24	DS	0.09	Class 5	Bad
	16	0	LDS	0.78	Class 2	Good
	17	0	LDS	0.8	Class 1	Very good

**Table 5:** Correlation among biotic metrics and physico-chemical parameters using Spearman correlation

Parameters	HBI	SIG	MAR	SHA	TNT	TNI	NoE	NoP	NoT	NEPT	NoI
Depth	−0.05	−0.49*	0.36	−0.09	−0.08	−0.51*	−0.55*	0.18	−0.28	−0.36	−0.51*
DO	0.45*	−0.19	0.12	0.23	0.15	−0.21	0.49*	0.37	0.09	0.69*	0.50*
pH	−0.22	0.03	−0.07	0.32	0.26	0.04	−0.01	0.56*	0.29	0.14	0.04
EC	−0.24	−0.23	−0.16	0.06	0.01	−0.20	−0.19	0.49*	0.01	−0.08	−0.20
T	−0.23	−0.27	−0.05	−0.23	−0.61*	−0.28	−0.24	0.11	−0.22	−0.15	−0.28
Turbidity	0.10	0.28	0.25	0.56*	0.57*	0.26	0.34	0.58^*^	0.37	0.41	0.26
NO3	−0.57*	−0.09	−0.19	0.18	0.05	−0.12	−0.06	0.35	0.11	0.05	−0.12
NO2	−0.24	0.23	−0.09	0.30	0.30	0.25	0.23	0.57*	0.21	0.35	0.25
NH4	−0.12	0.44	0.11	0.69*	0.59*	0.40	0.29	0.76*	0.49*	0.56*	0.40
PO4	0.21	−0.23	0.28	−0.16	−0.03	−0.28	−0.48*	−0.14	0.08	−0.23	−0.28
Nutrient	−0.25	0.26	−0.07	0.36	0.36	0.26	0.22	0.58*	0.28	0.38	0.26
BOD	0.65*	−0.36	0.25	−0.24	−0.20	−0.36	−0.33	−0.21	−0.35	−0.38	−0.36
TSS	−0.51*	0.32	−0.25	0.34	0.27	0.35	0.43	0.46	0.38	0.44	0.35
Sediment	−0.01	0.09	−0.55*	−0.24	−0.16	0.08	0.03	−0.52*	−0.17	−0.01	0.08

Values with an asterisk indicate a significant correlation (|*r*| ≥ 0.5, *P* < 0.05)

The results of the abiotic and biotic assessment of the water quality at the different sites indicate that some land uses may have impacted the water quality of the Tajan River, although not much information on all land uses was available (see online resource 1 and 2 of the supplementary material). However, we tried to get a good distribution of the sites along the river, so we could analyze the effects of land uses on the evaluated parameters. Between site 1 and site 2, there was a big fish pond which may have released pollutants into the river, hence increasing the BOD and nutrients (Fig. 2). This release may have affected the biology which is supported by the decrease of the MMI from class 1 to class 3 within the short distance between these sites (Table 4). This result is a serious alarm for local managers to consider more strict control measures on the aquaculture activities. Manures, fertilizers, and feeds applied to ponds to enhance production may have an effect on water quality and habitat structure (Andrieu et al. 2015). Some agricultural farms are located between sites 2 and 3, which apparently hardly had an effect on the water quality as the MMI was approximately the same between the sites (Table 4). The fourth site was an LDS site with an expected class 1 for the MMI. Upstream of the fifth site, there is a sand-mining site which deposits suspended solids into the river, thereby being a possible cause for the decrease of the MMI from class 1 to 2. Sand mining affects water ecological quality through contamination with increasing dissolved and suspended materials. Perhaps, the most common surface water contaminant is sediment or suspended solids. Sediment can smother the beds of receiving streams and directly affect benthic organisms (Swer and Singh 2004). Low pH, high EC, high concentration of ions of sulfate and iron and toxic heavy metals, low dissolved oxygen (DO), and high BOD were reported, which characterize the degradation of water quality (Saviour 2012).

The moderate MMI class (class 3) was detected at site 6 at which more people are settled due to the availability of good agricultural land, which may have impacted the water quality although the decrease in MMI between sites 5 and 6 is marginal (Table 4). Rice farms are located between sites 7 and 8, and the MMI showed that this land use may have decreased the water quality from the best class to class 2. Agriculture often results in increased nutrient levels and turbidity due to the use of fertilizers and erosion due to planting and harvesting. The effects from rice farms may be more important for the water quality than other farms (i.e., wheat farms or grain farms) because they require a lot of water for cultivation. Many scientists studied the effect of agriculture as a non-point pollutant on river basin and biota (Smakhtin 2002; McCarthy and Johnson 2009). The traditional agriculture present in the research area uses much water, and the effluent of the farms often runs directly into the river. Other selected sites were below the dam of the Tajan River, and their MMI are different from those upstream (Table 4). The dam sedimented the suspended solids and altered the physico-chemical parameters. Effects of dams on rivers are well documented including decreasing pollutants such as suspended solids and nutrients. However, there are also negative effects of dams on the ecosystem documented (Caudill et al. 2007). After the dam, site 9 had a high quality (class 1) which is comparable with an LDS. Between sites 9 and 10, there was a collection of three fish ponds that take up high volumes of clean water from the river and release the same volume back to the river downstream. This activity may have resulted in the lower MMI class at site 10 (Hering et al. 2006). Downstream of the fish ponds, the land is used for rice cultivation by the local people. This land use may have increased the pollution especially in suspended solids and nutrients, thereby decreasing DO and changing the ecological water quality at site 11 to a moderate quality (class 3) (Fig. 2). For a better understanding of the negative effects of the traditional agriculture on the Tajan River, see Abbasian et al. (2012) and Ahmadi-Mamaqani et al. (2011). Site 12 was located below the point where a small muddy stream, which is affected by sand-mining activities, joined the main river. A major component of the human use of aquatic systems is the construction, maintenance, and use of roads that occur as part of human infrastructure, and the road/stream interface is one of the main pathways for sediment to reach waterways. Stream crossings, often culverts, can alter in-stream sediment accumulations and geomorphology of a stream. The effects of sedimentation on macroinvertebrates have been well documented (Ogren 2014), as well as the effects of traffic, delving of bottom sand in rivers which may change the riparian zone of the site and may cause a decrease in the habitat quality and physico-chemical parameters. Sites 13 and 14 were chosen to show the effect of sand mining that was expressed by difference in levels of MMI. However, sand mining located between the sites as point source pollution may have increased the TSS and negatively affected the physico-chemical parameters, herewith affecting some sensitive species of macroinvertebrates. The sensitive species of macroinvertebrates are considered in the SIGNAL index (Chessman et al. 1997). Between sites 14 and 15, there was a pulping and papermaking factory that affected the river condition which has been documented already by Aazami et al. (2015). However, because of the plant’s wastewater, the MMI well showed that there were large differences in MMI between site 15 and the others sites (Table 4), especially compared with the LDS with same elevation (16, 17). pH and DO decreased and water temperature, BOD, and nutrients increased markedly between sites 14 and 15 (Fig. 2), resulting in the lowest MMI class for the sites 14 and 15 (the lowest class, bad). As expected, the MMI showed class 2 and 1 for sites 16 and 17, respectively. These sites were far away from human settlements and there was no pollution present. Finally, the MMI showed that all classes were found in the Tajan River (from very good to bad condition). On average, the MMI of Tajan River was higher in the upstream sites than that in the downstream ones (Table 4). Like Yazdian et al. (2014), we conclude that the overall MMI index worked well for our example river and could work in other Iranian regions, as well as it also provided a good assessment in Vietnam (Nguyen et al. 2014). Apparently, the MMI index is not so sensitive to differences in climate, biodiversity, physico-chemical parameters, and land uses. Aazami et al. (2015) showed that a classification based on biotic indices calculated from fish and macroinvertebrate abundance values did provide a better classification of the long-term environmental condition better than those based on abiotic indices.

### Biological Traits

**Fig. 3:**
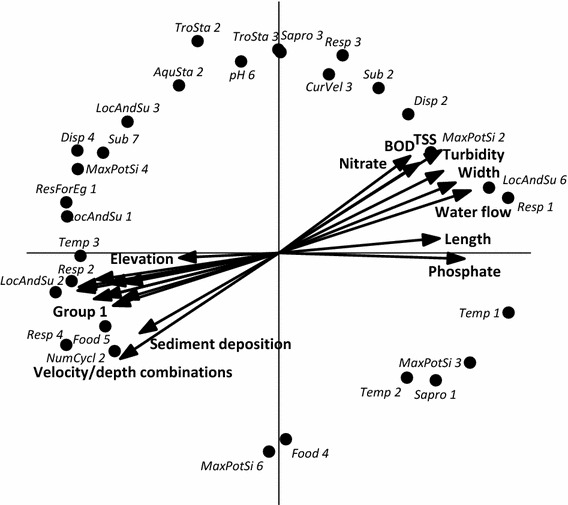
Redundancy analysis (or PCA, since the explanatory variables explain all variance) biplot showing the variation between the modalities of trait characteristics of macroinvertebrates and the correlation with physico-chemical parameters in Tajan River. Of all variance, 26 % is displayed on the horizontal axis and another 20 % on the vertical one. Only the 30 traits which had the strongest correlations with the physico-chemical parameters are shown. See Table 2 for trait abbreviations. Group 1 constitutes the habitat parameters bank vegetative protection, epifaunal substrate/available cover, embeddedness, channel flow status, channel alteration, frequency of riffles, bank stability, and riparian vegetative zone width as well as the sum of all habitat parameters

The variation between the modalities of trait characteristics of macroinvertebrates and the correlation with habitat and physico-chemical parameters is shown in Fig. 3. Twenty habitat and physico-chemical parameters have been shown to have a significant correlation with the trait composition of the macroinvertebrate communities present at the different sites (Fig. 3). The RDA biplot shows one gradient from the lower left quadrant to upper right quadrant with 10 parameters indicative of good habitat quality located in the lower left quadrant together with elevation, while some nutrient, physical measurements associated with large rivers, BOD, and turbidity are located in the upper right quadrant. In the lower left quadrant traits associated with having a large temperature tolerance, using gills or spiracles for respiration, being a surface swimmer, using macrophytes as food, and having 1 life-cycle per year are positioned close to the habitat parameters, while traits described as having an active aquatic dispersal, being relatively small, being present in interstitial water, and using teguments for respiration are positioned near the physico-chemical parameters in the upper right quadrant.

Griffith et al. (2001) also used RDA to assess the relationships among chemical and physical characteristics and macroinvertebrate assemblages at stream sites. Their results showed a close correlation among macroinvertebrate assemblages with water temperature and habitat-related parameters like mean substrate embeddedness and mean canopy density (Griffith et al. 2001). The same applies for the data set described in this paper which shows typical traits that are positively associated with good habitat quality like high DO (using gills or spiracles for respiration), availability of high-quality habitat (using macrophytes as food), and low disturbance (having 1 life-cycle per year) (Fig. 3). Unfortunately, like in many studies, the pollution gradient is collinear with elevation, so it is impossible to disentangle the effects of being an upstream site and being relatively clean. Parameters indicative of lowland rivers like width and depth are pointing in the biplot in the same direction as those indicative of the different impacts of land uses on environmental variables in Tajan basin (Fig. 3). In aquatic ecosystems, BOD is a symbol of pollution and it showed a correlation with nutrients like nitrate and phosphate (Llja et al. 2006). Figure 3 shows that traits which facilitate recovery (active aquatic dispersal), being able to cope with high flow (being present in interstitial water), and being able to cope with low DO levels (using teguments for respiration) are positively correlated with BOD and nutrients. These findings show that traits can be indicative of different kinds of stress but that more effort has to be put to gather data sets to disentangle the effect of habitat quality, pollution, and the physico-chemical properties of high- versus lowland rivers.

## Conclusion

Nowadays, the use of aquatic organisms as bio-indicators has been developed to assess ecological water quality in western regions, but have hardly been used in an Iranian context. We, therefore, evaluated the suitability of an up-to-date index (MMI) to assess the water quality for an Iranian case study. Unfortunately, based on MMI, some sites of the case study were indicated to have a very bad condition. Especially, the downstream sites are affected by food producing and industrial activities. These results are important for local managers of the studied river as well as those of other rivers in the north of Iran which are under stress of the same land uses. Monitoring and assessment tools for the management of water resources are generally more effective if they are based on a clear understanding of the mechanisms that lead to the presence or absence of species groups in the environment. We believe that our example shows that traits-based approaches are a useful tool to get a more mechanistic understanding of stressor–biology relationships.

## Electronic supplementary material

Supplementary material 1 (DOC 6719 kb)
