# Supplementary material for: Assessment of Ecological Quality of the Tajan River in Iran Using a Multimetric Macroinvertebrate Index and Species Traits
Source: Environ Manage. 2015 Apr 11;56(1):260–9. doi: 10.1007/s00267-015-0489-x (PMC4451864; doi:10.1007/s00267-015-0489-x)

**Environmental Management**

**Supplementary material to the paper:**

**Assessment of ecological quality of the Tajan river in Iran using a multimetric macroinvertebrate index and species traits.**

Jaber Aazami^1^, Abbas Esmaili Sari^1^, Asghar Abdoli^2^, Hormoz Sohrabi^3^ and Paul J. Van den Brink^4,5^

^1^ Department of Environment, Faculty of Natural Resources, Tarbiat Modares University, Tehran, Iran

^2^ Department of biodiversity and Ecosystem Management, Environmental Research Institute, Shahid Beheshti University, Tehran, Iran

^3^ Department of Forestry, Faculty of Natural Resources, Tarbiat Modares University, Tehran, Iran

^4^ Department of Aquatic Ecology and Water Quality Management, Wageningen University, Wageningen University and Research centre, Wageningen, The Netherlands.

^5^ Alterra, Wageningen University and Research centre, Wageningen, The Netherlands.

Corresponding author: [paul.vandenbrink@wur.nl](mailto:paul.vandenbrink@wur.nl); fax: +31-317-419000

Online resource 1: Tajan river sampling sites: geographic coordinates.

| Site | Latitude | Longitude | Altitude (m) | Width (m) | | Depth (m) | | Reason for selection |
| --- | --- | --- | --- | --- | --- | --- | --- | --- |
|  |  |  |  | Mean | SD | Mean | SD |  |
| 1 | 71 26 08 | 40 04 82.4 | 800 | 7.90 | 0.05 | 0.38 | 0.02 | Not affected by human activity. |
| 2 | 71 20 59 | 40 05 26.0 | 760 | 15.10 | 0.04 | 0.31 | 0.02 | 15 m after a trout farm effluent. |
| 3 | 70 92 38 | 40 06 75.1 | 695 | 16.50 | 0.09 | 0.30 | 0.03 | Draining the wastewaters of Siah-Dasht and Korcha residential area beside agriculture farms. |
| 4 | 70 81 89 | 40 01 14.5 | 802 | 6.90 | 0.13 | 0.36 | 0.05 | Not easy accessed and was chosen as Least-distributed site like site 1. |
| 5 | 70 77 69 | 40 06 59.5 | 639 | 7.20 | 0.11 | 0.32 | 0.04 | Draining of the agricultures wastewaters. |
| 6 | 70 61 66 | 40 10 26.0 | 532 | 19.50 | 0.09 | 0.42 | 0.02 | Assessing of jointed two upstream. |
| 7 | 72 66 06 | 40 07 09.1 | 1018 | 6.70 | 0.10 | 0.32 | 0.02 | Not affected by human activity. |
| 8 | 70 75 56 | 40 13 82.4 | 490 | 18.00 | 0.99 | 0.30 | 0.02 | Draining of the wastewater of rice farms. |
| 9 | 70 01 52 | 40 14 33.8 | 388 | 17.50 | 2.13 | 0.30 | 0.09 | Assessment of Sh-rajaei Dam. |
| 10 | 69 98 57 | 40 15 15.1 | 371 | 17.00 | 1.02 | 0.28 | 0.07 | 60 m downstream a trout farm effluent, and at a distance of approximately 1 km from dam. |
| 11 | 69 58 82 | 40 25 07.1 | 233 | 16.75 | 1.08 | 0.22 | 0.07 | Before of turbid effluent from Zellem sand mining. |
| 12 | 69 54 29 | 40 26 31.9 | 230 | 20.20 | 3.18 | 0.32 | 0.04 | After of turbid effluent from Zellem sand mining for showing the impacts. |
| 13 | 68 91 55 | 40 37 62.4 | 113 | 22.00 | 3.99 | 0.31 | 0.02 | Before major sand mining. |
| 14 | 68 86 94 | 40 38 04.0 | 110 | 22.70 | 2.87 | 0.32 | 0.01 | After of Major sand mining and also, before of wood and paper factory effluent. |
| 15 | 68 68 89 | 40 39 97.8 | 91 | 23.09 | 3.09 | 0.34 | 0.05 | After of wood and paper factory effluent with a distance about 500 m. |
| 16 | 68 51 12 | 40 33 64.0 | 177 | 7.65 | 0.42 | 0.09 | 0.10 | Not affected by human activity in downstream. |
| 17 | 68 95 92 | 40 23 52.6 | 407 | 5.60 | 0.73 | 0.06 | 0.13 | Not affected by human activity in downstream. |

Online resource 2: The land-use map of Tajan basin, Mazandaran province, north of Iran.


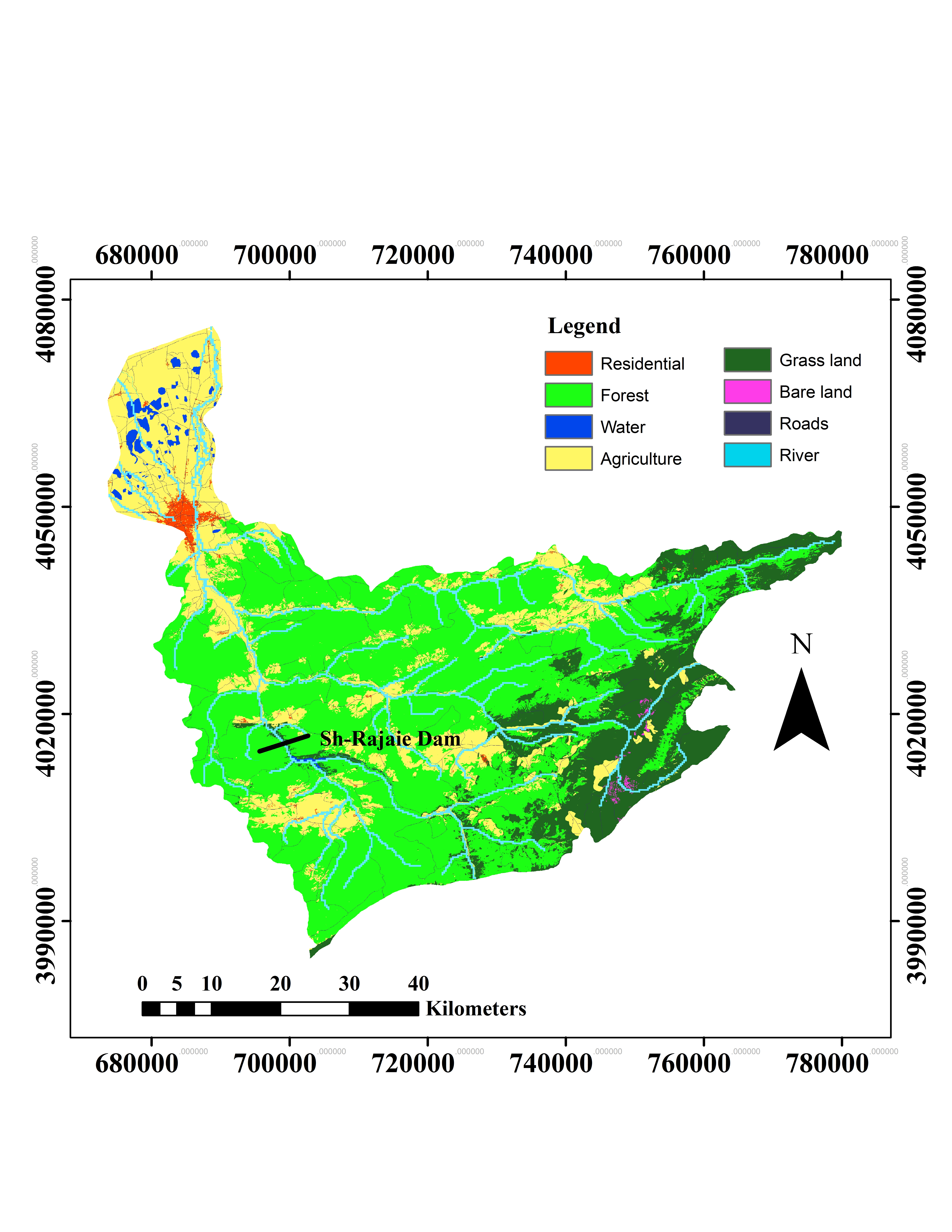

Supplement: Supplementary file 1 — Supplementary material 1 (DOC 6719 kb) [file 267_2015_489_MOESM1_ESM.doc]
